# Environmental Factors and Ventilation Affect Concentrations of Microorganisms in Hospital Wards of Southern Thailand

**DOI:** 10.1155/2020/7292198

**Published:** 2020-06-08

**Authors:** Nutthajit Onmek, Jinda Kongcharoen, Ailada Singtong, Angkana Penjumrus, Siripich Junnoo

**Affiliations:** ^1^Faculty of Science and Technology, Bansomdejchaopraya Rajabhat University, Bangkok 10600, Thailand; ^2^Faculty of Science and Industrial Technology, Prince of Songkla University, Surat Thani Campus, Surat Thani 84000, Thailand

## Abstract

Hospitals tend to have high density of occupancy. Poor indoor environmental quality in hospital buildings can exacerbate the health problems of patients and also harm visitors and staff. This study investigated the environmental characteristics and ventilation affecting the concentration of microorganisms in multiple-bed hospital wards. The measurements were accomplished by using a biosampler and an open plate method at four wards, different positions of electric fans, and different times. Data were analyzed by *t*-test and MANOVA. The results revealed that the concentrations of airborne bacteria were higher than the concentrations of fungi. There were significant differences in the concentrations of bacteria and fungi between sampling times and between hospital wards (*p* < 0.05), while no difference was observed by positions of electric fans. Correlations between the concentrations and other environmental parameters indicate that temperature, number of occupants, and humidity were physical factors associated with the concentrations of microorganisms. In addition, mostly, Gram-positive bacteria were observed. This indicates the conditions in buildings in the tropical climate, and regular housekeeping of all room areas is needed to prevent the growth of airborne contaminants and the related risks to patients, visitors, and staff.

## 1. Introduction

Hospitals are dynamic environments exposed to various indoor and outdoor environmental sources and can support microbial survival and growth [1–3]. Characterizing the airborne microorganisms in hospital buildings is important. There is an opportunity to spread infections via air by coughing, conversation, laughing, or sneezing. Outbreaks of infectious diseases, such as tuberculosis, measles, and influenza, have been linked with poor microbiological quality of hospital indoor air [4–6]. The presence of nosocomial infectious microorganisms is a source of nosocomial infections in hospitals [7]. These conditions may negatively impact the health of those working in such buildings, and microbial contamination may cause diseases.

Several studies have shown that microbial contamination of indoor air in a hospital is mostly by bacteria and fungi. There is increasing evidence of high airborne concentrations of bacteria in hospitals of Singapore [8]. The wards are potentially risky places for human occupants in a hospital. Investigation of airborne concentrations in different wards of a hospital was carried out in Isfahan, and high levels of airborne bacteria were observed [9]. Previous studies have focused on airborne concentrations of bacteria, while the concentrations of fungi have received less attention. In Thailand, Srion and Nathapindhu [10] found bacteria on about half the sampled surfaces, in Nonsang and Nongbua Lamphu hospitals. Also, seven genera of bacteria and fungi were identified in the air of Khonkaen hospital [11]. The environmental characteristics affecting airborne concentrations have not yet been determined.

The presence of high concentrations of bacteria and fungi within hospital buildings is related to the number of occupants, their activities, and ventilation. Microorganisms that are major causes of illness had increased concentrations in the environment affecting visitors. The high concentration of bacteria or fungi in the air indicates poor ventilation [12, 13]. Proper air ventilation is one method that can reduce the air pollution and the airborne microorganisms in buildings [14,15]. The strategy in the design of the ventilation system of the building is to control the environmental parameters, including air movement, humidity, temperature, air velocity, and cleanliness [1,16]. Generally, the microbiological quality indoors was not higher than the air quality standards and not higher than the outdoors. It is important to reduce and control the concentrations of harmful microorganisms in the air, to ensure good air quality. Several studies [17–19] have indicated that an important aspect in the work environment is the efficiency of the ventilation system and suggested associations among sources, risk factors, and transmission of microorganisms in the hospital and that air cleaning could decrease the concentrations.

In southern Thailand, high temperature and humidity favor airborne microorganisms, and poor indoor air quality in hospitals may cause diverse healthcare-associated infections of patients and staff. Our prior indoor air quality (IAQ) studies have reported on concentrations of *Aspergillus* spp., *Cladosporium* spp., and *Penicillium* spp. in three schools. They indicated that these fungi comprise a risk factor at the school, potentially inducing asthma symptoms in the students [20]. Previous studies [13,21,22] have indicated that air pollution sources (e.g., volatile organic compounds (VOCs), nitrogen dioxide (NO_2_), radon, ventilation, formaldehyde, humidity, temperature, and biocontaminants) might have negative effects on indoor air quality in schools. These studies suggested that the importance of natural ventilation could be focused and increased for improving indoor air quality and children's health. Schools, like hospitals, as communal premises, are critical environments due to the long stay and exposure of vulnerable groups of people. Thus, indoor air quality represents an important issue in both hospitals and schools due to the highest risk of exposure to contaminants, which can accumulate particularly when correct air ventilation is not implemented. Prasomwong et al. [23] sampled a hotel in Thailand, and the measurement results showed that humidity and temperature were both above recommended limits, and poor air quality was detected. Fatigue and general malaise, nasal symptoms, and eye irritation typical in sick building syndrome were found in hotel staff [24]. It can be seen that air quality and air quality management are related to diseases and their symptoms.

There are several factors involving indoor air quality. The environmental factors such as bioaerosol and human activity are also significant. Additionally, based on the risk assessment report of hospitals, the source of chemical emissions from wards is not considered. The study of relevant factors of indoor air quality management in hospitals is one approach to reduce hospital infections. Therefore, this current study aimed at determining the airborne contamination levels in multiple-bed wards of hospitals, where more stringent hygiene standards are pursued. The purposes of this study were to determine concentrations of airborne microorganisms within a large hospital center in southern Thailand. This study also compares the concentrations in different wards, relative to positions of electric fans, and by sampling time. Moreover, the physical environmental characteristics (temperature, humidity, and air velocity) and number of occupants expected to affect the concentrations of microorganisms were investigated.

## 2. Materials and Methods

### 2.1. Sample Location

The hospital assessed in this study has a large ward capacity with 800 beds in total, and controlled natural ventilation includes windows, doors, and electric fans. Gola et al. [25] informed that natural ventilation was identified as a strategy of healthier ventilation and natural force of wind-driven pressure through doors, apertures, and windows. The ventilation system of the ward was provided with wall fans for generating air motions. There were 18 electric fans in each ward, with ambient air at 25.4–30.3°C and 72% relative humidity. The four wards were selected for male surgery (A), female surgery (B), male medicine (C), and female medicine (D) with 40 beds. The service hours were from 12:00 am to 8:00 pm. Sampling was done in three daily periods: before (10:00–12:00 am), during (4:00–6:00 pm), and after (8:00–10:00 pm) the patient visits.

### 2.2. Sample Collection

**Figure 1:**
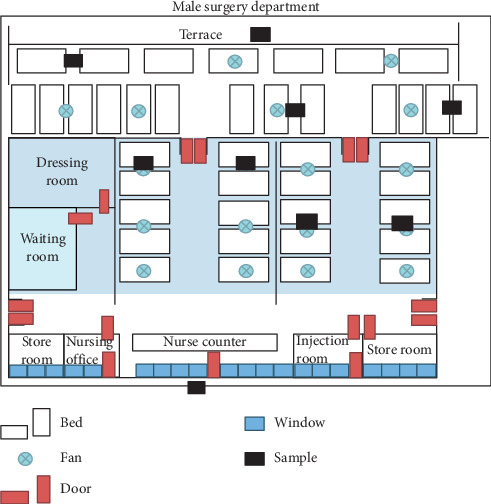
Sketch and the measurement in the multiple-bed ward.

This study was performed from June to October, which was rainy season, in a hospital. Airborne microorganisms were collected in 3 points relating wind turbulence from electric fans [26,27] to the positions of ceiling fans in the wards: under a ceiling fan, in the blow of a ceiling fan, and far away from the blow of ceiling fans (see Figure 1). The sampling methods were bioaerosol sampling and open plate method. Each position was measured as follows: bioaerosol sampler at 60 cm height from floor level was set up to collect 100 liters and an open plate with culture medium was put at 60 cm height from floor level for 20 minutes.

Trypticase Soy Agar (TSA) was used to cultivate bacteria and Malt Extract Agar (MEA) was used to cultivate fungi. The measurement of temperature and ventilation used a thermoanemometer (Model DF618) and WBGT Heat Stress Monitor was used to measure heat and humidity during each sampling period. The occupant density at the hospital was determined by observing the number of people within a period. The plates were incubated at 37°C for 24–48 hours and 48–72 hours for bacteria and fungi, respectively. Colony-forming units (CFUs) were counted and stained to identify the bacteria as Gram-positive or Gram-negative and to assess the morphology of microorganisms.

### 2.3. Data Analysis

The airborne microorganism levels were analyzed from descriptive statistics and with *t*-tests. Multivariate analysis of variance (MANOVA) was carried out to explore group differences among the concentrations of bacteria and fungi by section wards, positions of electric fans, and time. When differences were detected, post hoc comparisons were performed using Tukey's honestly significant difference (HSD) to test the difference between different pairs of the groups [28]. Differences were considered significant for *p* < 0.05. The associations among environmental characteristics with concentrations of bacteria and fungi were assessed from Pearson's correlation coefficients.

## 3. Results and Discussion

### 3.1. Concentrations of Bacteria and Fungi by Hospital Wards and Various Times

**Figure 2:**
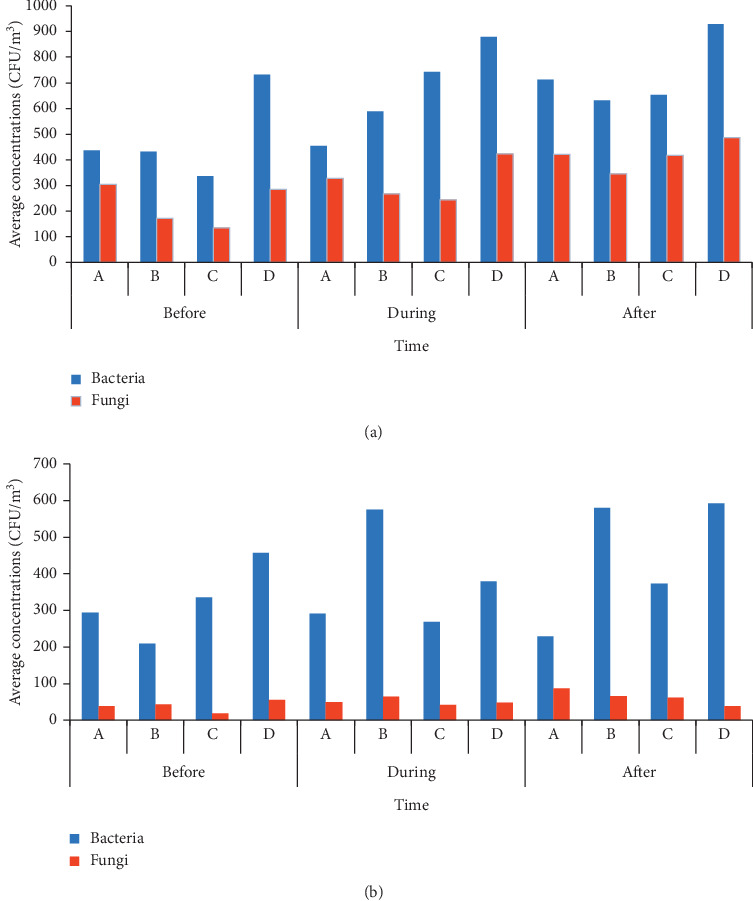
Bacterial and fungal concentrations in four hospital wards at three daily periods, measured using (a) biosampler and (b) open plate method.

The average concentration of bacteria in bioaerosols sampler ranged from 335.5 to 928.89 CFU/m^3^ whereas the open plate had the range 209.69–592.06 CFU/m^3^. The highest average concentrations of bacteria were detected after the visiting hours in ward D, according to both measurement methods (see Figure 2). Regarding the fungal concentrations, the average concentration from bioaerosols sampler method ranged from 134.50 to 487.22 CFU/m^3^, and for open plate measurements, the range was 18.82–87.50 CFU/m^3^. Also, the highest average concentrations of fungi were found in ward D when using bioaerosols sampler, but in ward A when using open plate at nighttime. Airborne microorganisms reached their concentration peak after the patient visits and then gradually decreased until the next visits.

Our study revealed that the concentrations of airborne bacteria were higher than those of fungi, according to both sampling methods, agreeing in this sense with a prior study [29]. Augustowska and Dutkiewicz [30] indicated as monthly averages, measured in a hospital ward in Poland, 257–436 CFU/m^3^ of bacteria and 10–96 CFU/m^3^ of fungi. Our measurement results showed higher airborne concentrations than this prior study of a hospital ward in Poland. However, the measurement results of concentrations of bacteria did not exceed the standard limit of NIOSH set in 2004 and ACGIH (1,000 CFU/m^3^), and the fungal concentration did not exceed the 500 CFU/m^3^ of fungi guideline set by WHO in 2000 [31,32]. Higher airborne concentrations during nighttime were observed after the visiting hours (i.e., after 8:00 pm). Most microorganisms tend to come from skin (exudate) infected lesions and dust from human respiration [33], so the concentration of microorganisms tends to be higher indoors than outside of the building. Previous studies have reported greater concentrations indoors than outdoors [31], and our study agrees with this pattern. Ekhaise et al. [7] and Jaffal et al. [34] concluded that the indoor air environment can potentially place patients at greater risk than the outside environment because enclosed spaces can confine aerosols and allow their accumulation to reach infectious levels. The hospital wards have no housekeeping in the room areas after the service hours. This may increase infection risks for the patients. The level of cleanliness in the wards was considered.

### 3.2. Comparisons of Bacterial and Fungal Concentrations by Hospital Ward and by Sampling Time

The compared differences of bacterial and fungal concentrations at four different wards were statistically significant (*p* < 0.05), and the results are presented in Table 1. The results from biosampler indicate that the A, B, and C wards did not significantly differ in the mean bacterial concentrations, while ward D had significantly higher mean bacterial concentration than these (Table 2). For fungi, wards A and D had significantly higher mean concentrations than wards B and C. The open plate sampling gave no significant differences in the mean concentrations of bacteria at B and D or between A and C. In the fungal concentrations, A and B did not differ, and neither did C and D. This is because of the same department on the same floor of the hospital, as well as proper ventilation systems.

There were significant differences in the mean fungal concentrations between the time periods (*p* < 0.05). The highest concentrations were measured during and after patient visits. Both bacterial and fungal concentrations were comparatively lower before the visiting time than at other observation periods (Table 3). This might be because of the low night temperatures and high humidity. The high concentrations during and after visits could alternatively be attributed to the visitors. The results showed that there was no significant difference between the bacterial and fungal concentrations and the positions of electric fans. This study also revealed that the occurrence of air fluctuations and turbulence from electric fans did not affect exposure levels. The results disagree with the findings of a previous study [26] claiming that microorganism contamination increased with the ambient air exchange rate when the fan blew air downwards. Air quality inside was very similar to air quality outside, indicating that there was good natural ventilation quality implemented for the building.

### 3.3. Environmental Characteristics and Concentrations of Airborne Bacteria and Fungi

The average temperature, air velocity, and humidity given by biosampler were 27.068 ± 1.14°C, 0.84 ± 0.53 m/s, and 72.09 ± 0.287%, respectively, whereas the average temperature, air velocity, and humidity at the open plates were 27.21 ± 1.27°C, 0.67 ± 0.50 m/s, and 72.16 ± 0.66%, respectively. The high humidity, velocity, and air temperature exceed the indoor air quality standards by WHO and ASHRAE 55 [35, 36]. A summary of the concentrations and environmental characteristics is shown in Tables 4 and 5.

Concentrations of airborne bacteria and fungi were positively linked with the number of people in the ward (Table 6). Temperature and humidity affect fungal concentrations significantly. Using the open plate data, a significant negative correlation was observed between the fungal concentrations and temperature. In addition, fungal concentrations were positively correlated with humidity given by biosampler. Normally, fungi grow the best in warmth and high humidity [2]. In this study, patient rooms had a humid and warm climate, with a humidity of over 60% and a temperature of about 27°C. The high temperatures suggest that air conditioning was not much in use. It is probably too expensive, as it would be to dehumidify the air.

The number of occupants, humidity, and temperature are physical factors associated with bacterial and fungal concentrations; however, these were not significantly affected by air velocity. Previous studies have indicated that the density of occupants is the key factor influencing the airborne concentrations in hospital wards [7,33,37]. Our study agrees with these previous studies. Moreover, Zemouri et al. [38] demonstrated that the daily cleaning operations in the hospital were related to the presence of aerosols. Normally, this area was cleaned twice per day (in the morning and in the afternoon). This cleaning frequency may not be a sufficient standard for safety. The management of cleaning procedures and the awareness of cleanings should be considered. Furthermore, a correlation between the number of people and the air volume was reported [39]. Organizing patient visits in separate spaces, with distancing to prevent infections, and control of crowding seems reasonable. Further, for example, smartphones could be used for patient communications with family members during hospital visits. The results show that the fungal presence was very high, and aside from people, this may be attributed to air ventilation and air conditioning. Most of the bacteria related to patients can be released in a hospital environment and can be breathed in by visitors and staff [9,40]. This study contrasted with the findings on temperature by Park et al. [1] who reported that the temperature significantly influenced the concentration of bacteria. No relationships between bacterial or fungal concentrations and air velocity were found.

### 3.4. Concentrations of Gram-Positive and Gram-Negative Bacteria

Our results indicate that Gram-positive cocci and rod-shaped bacteria were present in significantly larger numbers than the Gram-negative bacteria throughout the experiment. It has been reported that Gram-positive bacteria are more tolerant to environmental stresses than Gram-negative bacteria. The cell walls of Gram-negative bacteria are thinner and less compact, whereas in Gram-positive cells peptidoglycan forms a thick compact cell wall, which is the outermost structure of Gram-positive cells [41]. Airborne Gram-positive cocci are most frequently detected indoors in hospitals [42]. The samples collected were analyzed by microscopy. The overall culturable fungi were *Aspergillus* spp., *Penicillium* spp., *Cladosporium* spp., *Alternaria* spp., and *Curvularia* spp. *Aspergillus* spp. was the most frequently identified species in this study, and these fungal pathogens are associated with airway diseases [43]. It has been reported that *Aspergillus* spp. are the most common fungi found in various environments, such as food, water, and plants. Warmth with high moisture and lack of sunlight is favorable for their growth [44,45]. A previous study reported that *Aspergillus* spp. caused aspergillosis and allergic aspergillosis syndrome [11,46–48]. A study on concentrations of airborne microorganisms indicated that most fungi were found in a food center (*Penicillium* spp., *Aspergillus* spp., and *Cladosporium* spp.) [49]. However, these did not cause illnesses or health problems, possibly due to our immune system. Moreover, *Penicillium* spp. and *Aspergillus* spp. are common fungi found in buildings and are not considered pathogens. Nevertheless, these fungi might cause allergy, asthma, or respiratory illnesses. Hameed et al. [29] also reported on the diversity of airborne bacteria and fungi in Helwan city of Egypt, where the fungi most found were *Aspergillus*, *Cladosporium*, and *Penicillium*, in this order. In addition, *Aspergillus* spp., *Penicillium* spp., *Cladosporium* spp., *Curvularia* spp., and *Alternaria* spp. were found indoors as microbiological contaminants in the air, at a university building [50]. Our results overall agree with these prior studies. Some particular fungi are linked with the sick hospital syndrome, causing allergenic diseases nonspecifically, and come from construction dust or dust accumulated within ventilation [50–53]. Most invasive infections are acquired from indoor air [54]. The prevention and control of microorganism growth can reduce the human health risks from airborne microorganisms. To reduce concentrations of airborne contaminants, it is necessary to implement some control measures in wards, such as using air purifiers and sterilization using ozone.

## 4. Conclusions

Based on our measurements in a large hospital, located in southern Thailand, the airborne bacterial concentrations were significantly higher than the fungal concentrations. The general air quality characteristics indicated that bacteria can grow and are more prevalent than mold. The airborne bacterial and fungal concentrations did not exceed the standard limits, respectively, set by the NIOSH and the WHO. This study also revealed that the occurrence of air fluctuations and turbulence from electric fans had no effects on the level of exposure. Air quality inside is very similar to air quality outside, indicating good natural ventilation quality in the building. The five types of fungi found were *Aspergillus* spp., *Penicillium* spp., *Cladosporium* spp., *Alternaria* spp., and *Curvularia* spp. In general, these fungi are mostly observed in high-risk areas such as schools, theatres, and hospitals. Exposure to these contaminants may affect the respiratory system. The results suggest that the number of occupants in a building is positively associated with the airborne bacterial concentrations. Additionally, high humidity and high temperature were observed in multiple-bed hospital wards. This might be because of the tropical climate in the area of this study. The quality of air in the wards was better early in the day than later after the morning has passed. Room cleaning should be performed after patient visits, to decrease airborne bacteria. Cleaning and housekeeping of all room areas need to be regular to decrease the airborne contamination in all areas for patients, visitors, and staff. Nosocomial infections with indoor air quality in the buildings will be investigated in related future studies. In further research on factors that affect indoor air quality, chemical factors should be considered.

## Figures and Tables

**Table 1:** MANOVA of bacterial and fungal concentrations by hospital ward and by sampling time.

Method	Source	Concentration	SS	df	MS	*F*	*p*

Biosampler	Ward	Bacteria	4353266.40	3	1451088.80	13.761^*∗∗∗*^	≤0.001
		Fungi	774011.64	3	258003.88	6.107^*∗∗∗*^	0.001
	Time	Bacteria	2766032.18	2	1383016.09	13.116^*∗∗∗*^	≤0.001
		Fungi	1359200.96	2	679600.48	16.086^*∗∗∗*^	≤0.001

Open plate	Ward	Bacteria	2348144.91	3	782714.97	30.900^*∗∗∗*^	≤0.001
		Fungi	22250.63	3	7416.88	8.688^*∗∗∗*^	≤0.001
	Time	Bacteria	593514.98	2	296757.49	11.716^*∗∗∗*^	≤0.001
		Fungi	38179.84	2	19089.92	22.362^*∗∗∗*^	≤0.001

Note: ^*∗∗∗*^*p* < 0.001; SS: sum of squares; df: degrees of freedom; MS: mean square.

**Table 2:** Tukey's HSD post hoc test results by hospital ward.

Method	Concentration	A	B	C	D

Biosampler	Bacteria	533.81^a^	550.15^a^	577.00^a^	880.19^b^
	Fungi	351.19^ab^	261.36^a^	264.67^a^	318.70^b^

Open plate	Bacteria	271.56^a^	454.58^b^	325.69^a^	442.55^b^
	Fungi	40.69^a^	46.67^a^	57.40^b^	58.65^b^

Within each row, different letters indicate statistically significant differences, based on Tukey's HSD multiple comparison test.

**Table 3:** Tukey's HSD post hoc test results by time.

Method	Concentration	Before	During	After

Biosampler	Bacteria	479.59^a^	675.14^b^	749.05^b^
	Fungi	214.19^a^	309.86^b^	413.92^c^

Open plate	Bacteria	326.06^a^	376.89^b^	418.79^b^
	Fungi	38.79^a^	50.61^b^	62.50^c^

Within each row, different letters indicate statistically significant differences, based on Tukey's HSD multiple comparison test.

**Table 4:** Summary of the average bacterial and fungal concentrations and other key environmental characteristics by hospital ward.

Method		A	B	C	D	Recommended level

Biosampler	Bacteria (CFU/m^3^)	533.81	550.15	577.00	880.19	<1,000 FU/m^3^^*∗*^
	Fungi (CFU/m^3^)	351.19	261.36	264.67	318.70	<500 FU/m^3^^*∗∗*^
	Temperature (°C)	27.17^†^	27.09^†^	27.18^†^	26.83^†^	22–26.1°C^*∗∗∗*^
	Air velocity (m/s)	0.719^†^	0.832^†^	0.942^†^	0.817^†^	<0.25 m/s^*∗∗∗*^
	Humidity (%)	72.00^†^	72.33^†^	72.00^†^	72.00^†^	30–60% RH^*∗∗∗*^

Open plate	Bacteria (CFU/m^3^)	271.56	454.58	325.69	442.55	<1,000 FU/m^3^^*∗*^
	Fungi (CFU/m^3^)	40.69	46.67	57.40	58.65	<500 FU/m^3^^*∗∗*^
	Temperature (°C)	27.27^†^	27.36^†^	27.19^†^	27.05^†^	22–26.1°C^*∗∗∗*^
	Air velocity (m/s)	0.556^†^	0.698^†^	0.668^†^	0.741^†^	<0.25 m/s^*∗∗∗*^
	Humidity (%)	72.25^†^	72.05^†^	72.33^†^	72.00^†^	30–60% RH^*∗∗∗*^

Remark: ^*∗*^NIOSH, 2004; ^*∗∗*^WHO, 2000, ^*∗∗∗*^ASHRAE 55, 2004; ^†^not in compliance with standard.

**Table 5:** Summary of the average bacterial and fungal concentrations and other key environmental characteristics by hospital time.

Method		Before	During	After	Recommended level

Biosampler	Bacteria (CFU/m^3^)	479.59	675.14	749.05	<1,000 FU/m^3^^*∗*^
	Fungi (CFU/m^3^)	214.19	309.86	413.92	<500 FU/m^3^^*∗∗*^
	Temperature (°C)	27.662^†^	26.553^†^	26.99^†^	22–26.1°C^*∗∗∗*^
	Air velocity (m/s)	0.814^†^	0.808^†^	0.888^†^	<0.25 m/s^*∗∗∗*^
	Humidity (%)	72.00^†^	72.00^†^	72.27^†^	30–60% RH^*∗∗∗*^

Open plate	Bacteria (CFU/m^3^)	326.06	376.89	418.79	<1,000 FU/m^3^^*∗*^
	Fungi (CFU/m^3^)	38.79	50.61	62.50	<500 FU/m^3^^*∗∗*^
	Temperature (°C)	28.039^†^	26.580^†^	27.022^†^	22–26.1°C^*∗∗∗*^
	Air velocity (m/s)	0.701^†^	0.622^†^	0.678^†^	<0.25 m/s^*∗∗∗*^
	Humidity (%)	72.04^†^	72.18^†^	72.26^†^	30–60% RH^*∗∗∗*^

Remark: ^*∗*^NIOSH, 2004; ^*∗∗*^WHO, 2000, ^*∗∗∗*^ASHRAE 55, 2004; ^†^not in compliance with standard.

**Table 6:** Correlations between the concentrations and other environmental characteristics.

Factor	Biosampler		Open plate	
	Bacteria	Fungi	Bacteria	Fungi

Temperature	0.076	−0.012	0.045	−0.162^*∗∗*^
Number	0.405^*∗∗*^	0.461^*∗∗*^	0.121^*∗*^	0.123^*∗*^
Air velocity	0.006	0.068	0.029	0.049
Humidity	0.072	0.149^*∗*^	0.050	0.026

Note: ^*∗*^*p* < 0.05; ^*∗∗*^*p* < 0.01.

## Data Availability

The data used to support the findings of this study are available from the corresponding author upon request.
